# A Defined, Feeder-Free, Serum-Free System to Generate *In Vitro* Hematopoietic Progenitors and Differentiated Blood Cells from hESCs and hiPSCs

**DOI:** 10.1371/journal.pone.0017829

**Published:** 2011-03-18

**Authors:** Giorgia Salvagiotto, Sarah Burton, Christine A. Daigh, Deepika Rajesh, Igor I. Slukvin, Nicholas J. Seay

**Affiliations:** 1 Department of Research and Development, Cellular Dynamics International, Inc., Madison, Wisconsin, United States of America; 2 Department of Pathology and Laboratory Medicine, University of Wisconsin, Madison, Wisconsin, United States of America; University of Minnesota, United States of America

## Abstract

Human ESC and iPSC are an attractive source of cells of high quantity and purity to be used to elucidate early human development processes, for drug discovery, and in clinical cell therapy applications. To efficiently differentiate pluripotent cells into a pure population of hematopoietic progenitors we have developed a new 2-dimentional, defined and highly efficient protocol that avoids the use of feeder cells, serum or embryoid body formation. Here we showed that a single matrix protein in combination with growth factors and a hypoxic environment is sufficient to generate from pluripotent cells hematopoietic progenitors capable of differentiating further in mature cell types of different lineages of the blood system. We tested the differentiation method using hESCs and 9 iPSC lines generated from different tissues. These data indicate the robustness of the protocol providing a valuable tool for the generation of clinical-grade hematopoietic cells from pluripotent cells.

## Introduction

Human embryonic stem cells (hESCs) and human induced pluripotent stem cells (hiPSCs) have the ability to proliferate indefinitely in an undifferentiated state, and to differentiate to virtually all mature cell types found in the human body when induced with the appropriate combination of growth factors and cytokines. Pluripotent cells offer a powerful system to create *in vitro* models of human development and disease, provide a valuable source of large quantities of mature cell types of consistent quality and purity for drug discovery and testing, and have strong potential for clinical cell replacement therapies. The hematopoietic system is of particular interest for these applications due to the wide range of progenitor and mature blood cell types, which could be generated from pluripotent cells, and for the already available large amount of information on the development and characterization of these cells. Moreover, establishing a protocol to induce differentiation of hESCs into hematopoietic progenitors provides an easy approach to access to initial steps of hematopoiesis during human ontogeny, which occur in the first weeks of the developing embryo and are therefore impractical to study *in vivo*. Finally, a robust differentiation method together with the accessibility of patient-specific pluripotent cell lines provide a novel approach to study blood disorders [1], and generation of patient-specific multipotent hematopoietic progenitors could eventually be used in cellular therapy.

Despite the differentiation method used, *in vitro* hematopoietic differentiation from pluripotent cells (hESCs or hiPSCs) seems to progress through the same stages of hematopoietic development *in vivo*: during the initial week the differentiating hESC cultures are characterized by the presence of hemato-endothelial progenitors (hemangioblast) [2], [3]–[5]. This initial developmental stage *in vitro* appears to resemble the yolk-sac phase of hematopoiesis *in vivo*, when hematopoietic progenitors have the potential to give rise to primitive erythroid cells, megakaryocytes and macrophages. Only with an extended differentiation time are hematopoietic progenitors capable of maturing further, and therefore acquiring a broader developmental potential. However, current methods for hematopoietic differentiation of pluripotent stem cells rely on the use of serum, co-culture on stromal cell lines or the formation of embryoid bodies (EB) [2], [3], [6]–[12]. The poorly defined factors present in bovine serum, as well as in conditions when feeder cells are used, and the high variability of the embryoid body system prompted us to develop a new, defined, animal product-free differentiation system to generate clinical grade hematopoietic progenitors easily applicable to test the effect of small molecules in large scale screens. In the present study, we describe a novel 2-dimentional (2D), feeder-free, serum-free, highly efficient differentiation system for generating hematopoietic progenitors from hESCs. Our data also show the robustness of our protocol, as it induced the same pattern of hematopoietic differentiation observed from hESC in 9 hiPSC lines generated from different somatic cell types. In summary, a single matrix protein is sufficient to support hematopoietic differentiation using a cocktail of growth factors and hypoxic conditions that resemble the environment in the developing embryo.

## Results

### Effect of matrix proteins and oxygen concentration on hESC-derived hematopoietic progenitor development

**Figure 1:**
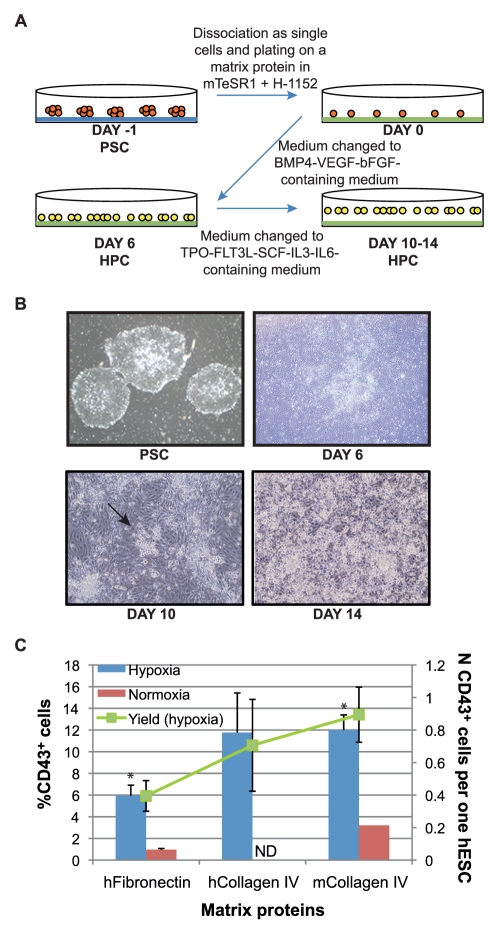
Optimization of the 2D differentiation protocol for hematopoietic differentiation. (A) The 2D differentiation protocol. Pluripotent stem cells (PSC) grown as colonies (in orange) on Matrigel (in blue) are dissociated and plated on a matrix protein (fibronectin or collagen IV, in green). After 24 hrs growth factors are added to the medium to induce hematopoietic progenitor cell (HPC, in yellow) development. The growth factor cocktail is change after 6 days of differentiation. (B) Cell morphology during the differentiation of pluripotent stem cells. After 6 days of differentiation, cells remain adherent, but change in morphology. Only after 10 days of differentiation do some colonies of round, loosely attached cells start to appear (arrow). After 14 days of differentiation many round, loosely attached cells cover the dish on top of a layer of adherent cells. (C) Percentage of CD43^+^ cells after 6 days of differentiation on different matrix proteins. Standard errors are indicated (Hypoxia in blue: n = 20 on hFibronectin, n = 3 on hCollagen IV, n = 27 on mCollagen IV; Normoxia in red: n = 5 on hFibronectin, n = 1 on mCollagen IV). Statistically significant difference between hFibronectin and mCollagen IV is indicated with a star (*) p<0.005. The yield of CD43^+^ cells (in green) is calculated as total number of CD43^+^ cells generated per each hESC induced to differentiate under hypoxic conditions.

To establish a completely defined 2D hematopoietic differentiation method, we tested the ability of matrix support proteins and altered atmospheric conditions to promote serum–free hematopoietic and endothelial differentiation from hESCs induced by a cocktail of growth factors (BMP-4, VEGF and bFGF) already shown to support hemato-endothelial differentiation from hESCs grown on MEFs (mouse embryonic fibroblasts) [13]. A schematic diagram of the differentiation protocol used in this study is depicted in Fig. 1A. We selected human fibronectin and collagen IV as matrix proteins because they are known to support hematopoietic differentiation of ESC-derived Flk1 progenitors in the mouse system [14]. Fibronectin has been implicated in the progression of mesodermal differentiation [15] and is commonly used to induce endothelial differentiation from endothelial progenitors (HUVEC or hESC-derived). Collagen IV has been reported to promote mesoderm development, as it has been used to induce endothelial, cardiovascular and hematopoietic differentiation from murine ESCs [16], [17], from murine iPSCs [18] and from human ESCs [19].

To mimic the environment in the growing embryo at the stage when hemato-endothelial progenitors start to develop, we examined the role of hypoxia (5% O_2_ tension) during hematopoietic commitment to reflect more physiological oxygen levels. Hypoxia has been shown to have an important role *in vivo* in the very early stages of the growing embryo [20]. Before the establishment of the cardiovascular system, mammalian development occurs in a 3% oxygen environment. This physiological hypoxia seems to be an important regulator of embryonic angiogenesis and hematopoiesis. Among other effects, this low oxygen concentration induces expression of genes (such as FLK1, BMP-4 and VEGF) and stimulation of pathways (hypoxia inducible factor, HIF-dependent pathways) required for the formation and proliferation of the hemato-endothelial progenitors [21]–[23]. Moreover, hypoxia has already been shown to promote endothelial differentiation from hESCs [24]. The presence of hematopoietic progenitors was evaluated after 6 days of differentiation by the expression of CD43, the earliest hematopoietic-specific marker shown to appear in the hESCs/OP9 co-culture differentiation system [5].

As shown in Fig. 1C, both matrix proteins favored the attachment of the hESCs and the subsequent hematopoietic differentiation, with a significantly higher number of hematopoietic cells generated on collagen IV, as compared to fibronectin at day 6. On both matrix proteins we observed an increased and more consistent efficiency of hematopoietic progenitor generation in hypoxic cultures, which was therefore the condition of choice for the following experiments. Since murine collagen IV promoted hematopoietic differentiation as efficiently as the human counterpart, all subsequent studies were performed using murine collagen IV. In order to have a completely defined protocol for generating hematopoietic progenitors, we tested two serum substitutes, containing human serum albumin, human recombinant insulin and human transferrin (HIT and Serum Replacement 3). In hypoxic conditions, both reagents sustained hematopoietic and endothelial differentiation from hESCs plated on collagen IV, as assessed by the presence of CD43^+^CD34^+^ hematopoietic progenitors and CD31^+^CD34^+^CD43^−^ endothelial cells after 6 days of differentiation (data not shown).

### Characterization of the hESC-derived hematopoietic progenitors

**Figure 2:**
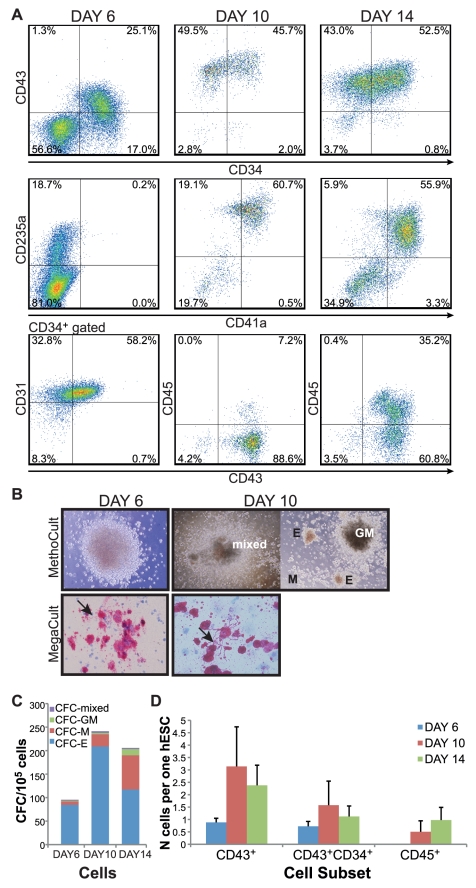
Characterization of the hESC-derived hematopoietic progenitors. (A) FACS analysis of differentiated cells at day 6 (total culture), at day 10 (only floating cells analyzed) and at day 14 (only floating cells analyzed). Representative FACS plots are indicated, showing live- and CD34^+^-gated events for CD43^+^CD31^+^ cells at day 6, and live-gated events for all the other plots. (B–C) Colony assays of hESC-derived hematopoietic progenitors harvested after 6 days (total culture) and after 10 or 14 days (floating cells) of differentiation. In methylcellulose-based assay (MethoCult), day 6 hESC-derived progenitors generated salmon-pink erythroid colonies; floating hESC-derived progenitors generated CFC-GM, CFC-M, CFC-E and mixed colonies. Average CFC values per 10^5^ cells ± standard errors: Day 6 N = 9 CFC-E 84.69±32.33, CFC-M 7.68±2.54, CFC-GM 1.49±0.93, mixed 1.58±0.32; Day 10 N = 5 CFC-E 208.88±75.59, CFC-M 26±11.90, CFC-GM 2.88±1.97, mixed 3.52±0.75; Day 14 N = 8 CFC-E 117.33±52.40, CFC-M 72.63±20.28, CFC-GM 13.28±4.36, mixed 2.5±0.98. In collagen-based assay (MegaCult), hESC-derived progenitors developed large colonies of megakaryocytes capable of shedding pro-platelets (arrow). (D) Yield of hematopoietic cell subsets obtained per one differentiating hESC at day 6 (in blue), day 10 (in red) or day 14 (in green). Standard errors are indicated (CD43^+^ cells: at day 6 N = 23, at day 10 N = 11, at day 14 N = 16; CD43^+^CD34^+^ cells: at day 6 N = 18, at day 10 N = 8, at day 14 N = 16; CD45^+^ cells: N = 23, at day 10 N = 6, at day 14 N = 11).

After 6 days the CD34 progenitor marker appeared on up to 40% of the hESCs differentiating on collagen IV under hypoxic conditions using serum-free substitutes (Fig. 2A). Early CD34^+^ cells are a heterogeneous population consisting of hematopoietic, endothelial and mesenchymal progenitor cells [8]. In our 2D culture system, after 6 days of differentiation almost every hESC generated one CD34^+^CD43^+^ hematopoietic progenitor (Fig. 2D). This population of CD34^+^CD43^+^ hematopoietic progenitors represented more than half of the CD34^+^ cells and 25% of the total culture (Fig. 2A). The optimized culture conditions also favored the generation of CD34^+^CD31^+^CD43^−^ endothelial progenitors (up to 50% of cells at day 6), consistent with the hemato-endothelial development in the early stages of hematopoiesis in the embryo. HESC-derived progenitors harvested after 6 days of differentiation gave rise to pink-colored erythroid colonies in serum-free methylcellulose-based colony assays (Fig. 2B). At this developmental stage, hematopoietic cells (CD43^+^) express the progenitor marker CD34 and the erythroid lineage marker CD235a (GlycophorinA) (Fig. 2A). Although we could not detect CD41a, a megakaryocyte marker, in these early differentiating cultures at day 6, these progenitor cells were able to generate megakaryocyte colonies in collagen-based colony assays (Fig. 2B), suggesting the presence of erythro-megakaryocyte progenitors, as it has been previously shown in the OP9 co-culture system [25].

**Figure 3:**
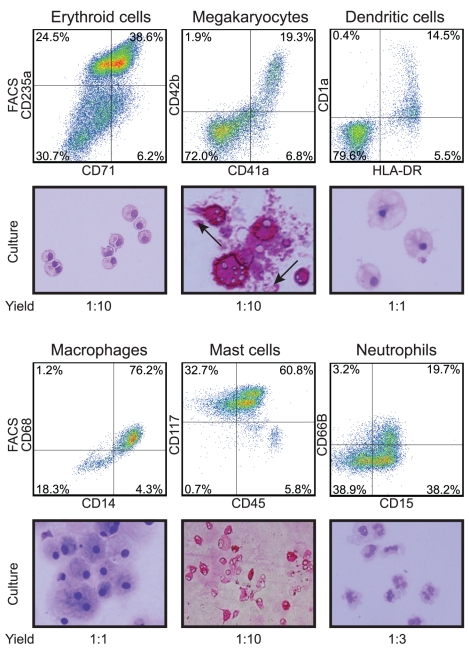
Characterization of the developmental potential of hESC-derived hematopoietic progenitors. FACS analysis and corresponding cytospins of mature cells generated in culture from hESC-derived progenitors induced with defined factors. Images were taken at 40× magnification. Cytospins of erythroid cells, dendritic cells, macrophages and neutrophils were stained with Wright. Cytospins of megakaryocytes were stained for GPIIb/IIIa (CD41a). Cytospins of mast cells were stained for tryptase. The yield hESC:mature cell indicates the number of each mature cell type generated per each hESC induced to differentiate.

Prolonging the differentiation resulted in the appearance of loosely attached, hematopoietic progenitor cells at around day 10 of differentiation (Fig. 1B). Collection of the floating cells in the culture resulted in an almost pure hematopoietic population consisting of 90% CD43^+^ cells (Fig. 2A), with average yield of three hematopoietic cells generated per hESC (Fig. 2D). Although the majority of these cells still consisted of erythro-megakaryocytic progenitors (80% of the CD43^+^ cells are CD235a^+^CD41a^+/−^), the pan-hematopoietic marker CD45 started to be expressed together with CD34 and CD43 (7% of the culture). CD34^+^CD43^+^CD45^+^ is a phenotypical signature for hematopoietic multipotent progenitors, as confirmed in methylcellulose-based colony assay where these cells were able to give rise to CFC-E, CFC-M, CFC-GM and mixed colonies, typical of multipotent hematopoietic progenitors (Fig. 2B, C). Extending the differentiation for 4 days expanded the CD45^+^ cells which represented 35% of the floating cells (Fig. 2A). At the end of this protocol the total yield of the CD45^+^ subset was of one CD45^+^ cell generated per hESC induced to differentiate (Fig. 2D). The multiple developmental potential of these cells was also confirmed in further differentiation cultures to mature cell types. Using different combinations of growth factors, from the hESCs-derived progenitor cells we were able to obtain CD71^+^CD235a^+^ erythroid cells, CD41a^+^CD42b^+^ megakaryocytes, HLA-DR^+^CD1a^+^ dendritic cells, CD14^+^CD68^+^ macrophages, CD45^+^CD117^+^ expressing tryptase mast cells and CD15^+^CD66B^+^ neutrophils (Fig. 3).

### Hematopoietic differentiation from 9 hiPSC lines generated from different tissues

**Figure 4:**
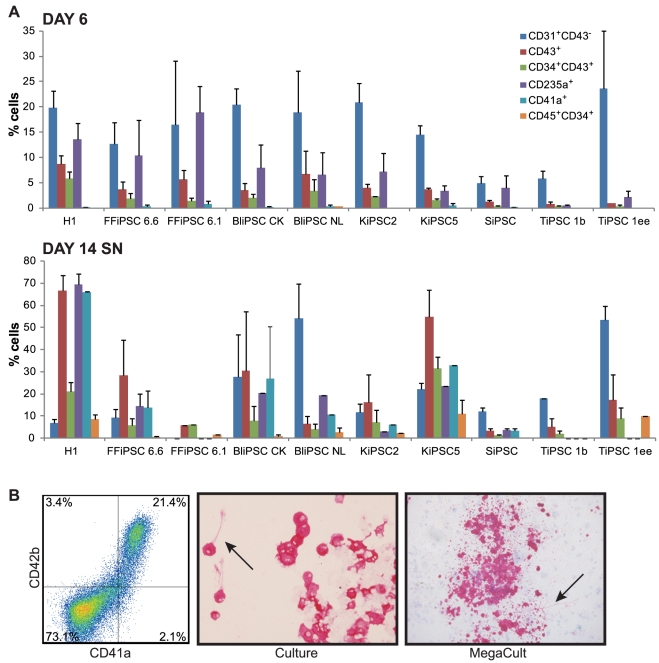
Characterization of the developmental potential of 9 hiPSC lines generated from different donor tissues. (A) Percentage of positive cells for the indicated marker in the differentiating culture. Total culture at day 6 of differentiation, or floating cells at day 14 were analyzed. Standard errors are indicated (n = 10 H1, n = 3 FFiPSC 6.6, n = 2 FFiPSC 6.1, n = 3 BliPSC CK, n = 4 BliPSC NL, n = 2 KiPSC2, n = 3 KiPSC5, n = 6 SiPSC, n = 2 TiPSC 1b, n = 3 TiPSC 1ee). (B) FFiPSC 6.1-derived hematopoietic progenitors isolated at day 10 of differentiation generated CD41a^+^CD42b^+^ megakaryocytes in culture, as indicated by flow cytometry analysis, cytospins stained for GPIIb/IIIa (CD41a), and megakaryocytes colonies in collagen-based assay. The megakaryocytes were able to produce pro-platelets (arrow) visible both in culture and in collagen-based colony assay.

The optimized 2D hematopoietic differentiation was tested on several hiPSC lines. The hiPSC lines were generated with viral transduction of human mature cells of different tissue origin: two lines were generated from human fetal fibroblasts (FFiPSC); two lines were generated from CD34^+^ PBMCs collected from a donor leukocyte pack (BliPSC); two lines were generated from keratinocytes isolated from primary human keratinocytes (KiPSC); one line was generated from adult skin fibroblast (SiPSC); two lines were generated from T-cells isolated from a donor leukocyte pack (TiPSC) [26]. We used the differentiation protocol optimized to achieve the maximum yield from the hESC line as standard conditions to compare all the hiPSC lines. After 6 days of differentiation we detected CD34^+^CD43^+^ hematopoietic and CD31^+^CD43^−^ endothelial progenitors from all iPSC lines (Fig. 4A). Similar to hESC-derived progenitors, hiPSC-derived hematopoietic progenitors harvested at day 6 appeared to be restricted to the erythroid lineage for the presence of the erythroid marker CD235a on the cell surface. These progenitors could generate megakaryocytes in colony assays as well as in culture, when induced with defined factors (Fig. 4B). For all the hiPSC lines, prolonging differentiation for 8 more days resulted in further development of the hematopoietic progenitors, which started to express CD45 while still retaining CD34 expression (Fig. 4A). The floating iPSC-derived hematopoietic progenitors were able to differentiate further into several lineages (Fig. S1) similarly to the hESC-derived progenitors. Despite the successful hematopoietic differentiation from all nine different hiPSC lines, we observed a high variability in differentiation efficiency. Variability in the efficiency of hematopoietic progenitor differentiation has been reported in an analogous analysis performed on different hiPSC lines using the OP9 co-culture system [11]. We could not observe any correlation between the hematopoietic differentiation efficiency and the cell origin of each hiPSC line. These differences might be either the result of viral integration in each clone, or simply an intrinsic variability among the lines, that has also been observed among hESC lines [11] and among iPSCs of murine origin [27]. We can therefore conclude that under the optimized condition for hematopoietic differentiation, we did not observe consistent differences between hESC and iPSC groups.

## Discussion

The present work outlines an efficient and directed differentiation method to generate hematopoietic progenitors from human pluripotent cells using a 2D, feeder-free, serum-free, completely defined system. For potential clinical applications of cells generated with the described method, we show that our system can be easily converted to xenogenic-free conditions, as the only reagents of non-human origin used for our differentiation protocol are the bFgf of zebrafish origin in the differentiation medium, and bovine serum albumin in serum replacer and in the pluripotent cell growth medium (mTeSR1), which can be substituted with the human counterparts. Other 2D methods shown to support hemato-endothelial differentiation include the use of mouse embryonic fibroblasts [13], bone marrow stromal cells, such as S17, MS-5 or OP9, [7], [8], or cells derived from fetal liver or AGM region of murine embryos [28]. The overall differentiation efficiency of these methods is quite low considering the fraction of CD34^+^ multipotent cells obtained in the culture (0.9%–20%), and even lower if the hematopoietic progenitors expressing CD45 are considered (0.1%–8%) during 7–20 days of differentiation. The only report on the use of collagen IV to sustain hematopoietic differentiation from hESCs does not provide information on the efficiency of the method, since the progenitors were not quantified [19]. The present protocol is completely defined and generates hematopoietic and endothelial cells at very high efficiency. The hypoxic conditions improved the percentage of hematopoietic cells after 6 days in culture using both fibronectin and collagen IV as matrix support proteins. Following 6 days of differentiation up to 40% of the cells expressed CD34 and up to 25% expressed CD43, generated with an average yield of one progenitor per hESC. After two weeks of differentiation we could isolate a large number of floating hematopoietic progenitor cells, of which 90% were CD43^+^ cells still retaining the CD34 marker and starting to express CD45 on their surface. Prolongation of the differentiation time also increased the yield of hematopoietic progenitors. We could show that this population of cells is able to differentiate further into mature cells of different lineages when specific growth factors for each lineage were added to the culture medium. Among the mature cell types we obtained using this method, of particular interest is the generation of megakaryocytes. To date, *in vitro* generation of megakaryocytes from human pluripotent cells has been largely achieved from somatic stem cells (bone marrow, or cord blood or mobilized peripheral blood CD34^+^ cells [29]–[35]). Recently, three groups have reported megakaryocyte differentiation from hESC lines using the murine OP9 co-culture system [25], [36], [37] and one group using the EB method [38]. Our work presents, therefore, for the first time a protocol devoid of serum and other undefined conditions to obtain megakaryocytes from hESCs and hiPCSs capable of shedding platelets *in vitro*. Obtaining large quantities of megakaryocytes *in vitro* could offer a valuable example of using hESC/hiPSC-derived cells to study disorders affecting a rare population of cells (megakaryocyte represent 0.1% of the nucleated cells in the bone marrow), but most importantly could set the stage for the production of a cell type, which could be used in clinical settings. It has been proposed that co-transplantation of autologous megakaryocytes together with hematopoietic stem cells could result in higher response and survival rates for patients afflicted by the severe thrombocytopenia often associated with high dose chemotherapy and radiation therapy [39]. The advantage of using autologous cells in transplantation studies prompted us to test our protocol on nine hiPSC lines generated in our laboratories using retroviral transduction of cells of different tissue origin. HiPSCs have already been shown to be able to differentiate into various lineages, such as cardiac [40], pancreatic [41], hepatic [42], epithelial [6], [43], [44], neuronal [45]–[47], adipose [48], and endothelial and hematopoietic [6], [11], [49]–[51]. The fast pace of basic research on hiPSCs since their discovery in 2007 [52], [53] reflects the high value of these new pluripotent lines for drug testing, preclinical models and clinical application. For the potential use of hiPSCs in pre- and clinical settings the major challenge is to define culture conditions to differentiate progenitor cells into a selected lineage with high efficiency and purity. Here we tested several hiPSC lines generated by our group for their ability to differentiate into hematopoietic progenitors using the 2D protocol optimized on a hESC line. Despite some expected differences in differentiation efficiency, we were able to generate hematopoietic progenitors from nine hiPSC lines derived from 5 different tissue types. As proof of principle we generated megakaryocytes from fibroblast (FFiPSC)-derived iPSCs with the same efficiency and characteristics as hESC-derived megakaryocytes. There are no previous reports in the literature to show the megakaryocyte potential of hiPSCs and the production of platelet-releasing megakaryocytes *in vitro*. The hiPSC-derived hematopoietic progenitors also had the potential to develop to cell types of other lineages of the blood system, such as erythroid cells, macrophages and dendritic cells. In our system we did not detect a lack of hematopoietic potential in iPSC lines generated from reprogrammed fibroblasts as it has been observed in [54], possibly due to an erase of the epigenetic memory as a consequence of prolonged culture *in vitro* as it has been suggested in [55].We can therefore conclude that, although further optimization for each hiPSC line seems to be required to achieve a maxim yield of hematopoietic differentiation from each line, hiPSCs and hESCs exhibit the same developmental potential *in vitro*.

In conclusion, we propose here a highly efficient new system to generate *in vitro* hematopoietic progenitors from hESCs and hiPSCs in conditions free of animal products and undefined components, which is easily applicable in basic research, drug discovery testing and clinical settings.

## Materials and Methods

### Pluripotent stem cell cultures

hESCs H1 (WA01) were obtained from WiCell Research Institute and different hiPSC lines were generated in house by retroviral expression of OCT4/SOX2/KLF4/MYC or OCT4/SOX2/Lin28/Nanog in different donor tissue samples as described elsewhere (patent application n. 20100041054 published on Feb. 18, 2010). The pluripotent stem cells were maintained in colonies on Matrigel (BD)- coated plates in mTeSR1 (BD) and dissociated with dispase (Invitrogen) as described in [56]. HESC H1 line was used within 33 to 53 passage numbers. FFiPSC lines were used within 30 to 40 passage numbers. BliPSC lines were used within 12 to 30 passage numbers. KiPSC lines were used within 20 to 30 passage numbers. SiPSC line was used within 40 to 50 passage numbers. TiPSC lines were used within 10 to 30 passage numbers. Cells were grown in 5% CO_2_ and regular atmospheric O_2_ concentration.

### Differentiation of hESC/hiPSCs

The protocol of hematopoietic differentiation from hESC/hiPSCs was established during the development of iCell™ endothelial cells (Cellular Dynamics International, Inc.). To initiate hematopoietic differentiation, hESC/hiPSCs grown on matrigel were dissociated with TrypLe (Gibco, Invitrogen) and transferred at 20,000 cells/cm^2^ onto 6-well plates pre-coated with 3 µg/cm^2^ human plasma fibronectin (Gibco, Invitrogen) or murine collagen IV (BD) in mTeSR1 medium (Stem Cell Technologies) supplemented with soybean trypsin inhibitor (Invitrogen) and an inhibitor of Rho-associated kinase (ROCK) as survival factor (H1152, Sigma). After 24 hrs, the TeSR1 medium is replaced with a differentiation medium containing IMDM (Invitrogen), BIT (bovin serum albumin, human recombinant insulin, human transferrin, Stem Cell Technologies), monothioglycerol (450 µM, Sigma), non essential aminoacids (0.1 mM, Invitrogen), L-glutamine (2 mM, Invitrogen), recombinant human BMP4 (50 ng/ml, R&D), recombinant human VEGF (50 ng/ml, Invitrogen) and recombinant zebrafish bFGF (50 ng/ml) made in house. For the humanized version we substituted BIT with HIT (Stem Cell Technologies) or Serum Replacer 3 (Sigma). After 6 days of culture the cells are dissociated with TrypLe and analyzed by flow cytometry. To extend the hematopoietic differentiation, after one week the cytokine cocktail in the differentiation medium changed to a medium containing heparin (5 U/ml, Sigma), TPO (25 ng/ml), human recombinant SCF (25 ng/ml), FLT3L (25ng/ml), IL-3 (10ng/ml), IL-6 (10ng/ml), all from Invitrogen. At day 10 or 14 the floating cells were harvested and analyzed using flow cytometry and colony forming assays using methylcellulose-based serum-free medium (MethoCult H4436, Stem Cell Technologies) and collagen-based megakaryocyte colony assay (MegaCult, Stem Cell Technologies) according to the manufacturer's instructions. All cultures were performed in hypoxic conditions (5% O_2_ concentration, balanced with nitrogen).

### Differentiation of hematopoietic progenitors into mature cell type

Erythroid cells: Cells harvested after 6 days of differentiation were transferred in low attachment plates to a medium containing SFEM (Stem Cell Technologies), heparin (5 U/ml, Sigma), TPO (100 ng/ml), human recombinant SCF (100 ng/ml), FLT3L (100 ng/ml), IL-3 (10 ng/ml), IL-6 (10 ng/ml), all from Invitrogen. After 4 days erythroid cells were expanded in SFEM medium containing 0.3% Excyte (Serologicals), Holo-Transferrin (1 mg/ml, Sigma), Hydrocortisone (1µM, Sigma), Insulin (20 ng/ml, Sigma), SCF (50 ng/ml, R&D Systems), EPO (2 U/ml, R&D Systems), IL-3 (5 ng/ml), IL-6 (10 ng/ml) and TPO (50 ng/ml, Invitrogen) for additional 2 weeks. Subsequently, IL-3, IL-6 and TPO were removed from the medium and the cells cultured for one week, then analyzed by flow cytometry for the expression of CD235a (glycophorin A) and CD71.

Megakaryocytes: Cells harvested at days 6, 10, or 14 of differentiation were transferred to low attachment plates in medium containing SFEM (Stem Cell Technologies), heparin (5 U/ml, Sigma), TPO (100 ng/ml), human recombinant SCF (100 ng/ml), FLT3L (100 ng/ml), IL-3 (10 ng/ml), IL-6 (10 ng/ml). After 10 days of culture the presence of megakaryocytes was assessed by FACS for the expression of CD41a and CD42b and cytospins of the culture were stained for GPIIb/IIIa (CD41a).

Dendritic cells: After 14 days of differentiation the floating hematopoietic cells in suspension were collected and expanded for a week in SFEM (Stem Cell Technologies), 1% Excyte (Serologicals), monothioglycerol (450µM, Sigma), non essential aminoacids (0.1 mM, Invitrogen), L-glutamine (2 mM, Invitrogen), GM-SCF (100 ng/mL, Leukine). The cells were then placed in dendritic cell differentiation medium containing SFEM, 1% Excyte, monothioglycerol (450µM, Sigma), non essential aminoacids (0.1 mM, Invitrogen), L-glutamine (2 mM, Invitrogen), GM-SCF (20 ng/mL), IL-4 (20 ng/ml, Peprotec), TNF-α (2.5 ng/mL, Peprotec). After one week the presence of dendritic cells was assessed by FACS for the expression of CD1a and HLA-DR.

Macrophages: After 14 days of differentiation the floating hematopoietic cells in suspension were collected and expanded for a week in SFEM (Stem Cell Technologies), 1% Excyte (Serologicals), monothioglycerol (450µM, Sigma), non essential aminoacids (0.1 mM, Invitrogen), L-glutamine (2 mM, Invitrogen), GM-SCF (100 ng/mL, Leukine). The cells were then transferred to macrophage specific medium: SFEM (Stem Cell Technologies), 1% Excyte (Serologicals), monothioglycerol (450 µM, Sigma), non essential aminoacids (0.1 mM, Invitrogen), L-glutamine (2 mM, Invitrogen), M-CSF (20 ng/mL, Peprotec), IL-1β (10 ng/mL, Peprotec). After one week the presence of macrophages was assessed by FACS for the expression of CD14 and CD68.

Mast cells: After 14 days of differentiation, cells were harvested and cultured for 14 days in low attachment plates in medium containing SFEM (Stem Cell Technologies), heparin (5 U/ml, Sigma), TPO (100 ng/ml), human recombinant SCF (100 ng/ml), FLT3L (100 ng/ml), IL-3 (10 ng/ml), IL-6 (10 ng/ml). Finally cells were transferred in medium containing StemPro-34 (Invitrogen), non essential aminoacids (0.1 mM, Invitrogen), L-glutamine (2 mM, Invitrogen), SCF (100 ng/ml, Invitrogen), IL-6 (100 ng/ml, Invitrogen). Mast cells were analyzed by FACS after three weeks in this last medium for the expression of CD117 and CD45, and the cytospins stained for Tryptase.

Granulocytes: After 14 days of differentiation on collagen IV, the cytokine cocktail was substituted with only G-CSF (100 ng/ml, Invitrogen) and after one week the presence of neutrophils was assessed by FACS for the expression of CD66B and CD15.

### Flow cytometry and immunostaining

All FACS antibodies CD34 FITC, CD43 APC, CD43 FITC, CD31 PE, CD235a PE, CD41a FITC, CD45 APC, CD71 FITC, CD42b APC, HLA-DR PE, CD1a APC, CD14 APC, CD68 PE, CD117 PE, CD66B FITC, CD15 APC were from BD Biosciences. CD68 staining was performed after cell permeabilization with Fix&Perm reagents (Caltag, Invitrogen). Cytospins of erythroid cells, dendritic cells, macrophages and neutrophils were stained with Wright (Protocol) according to manufacturer's instructions. Cytospins of megakaryocytes were stained for GPIIb/IIIa (CD41a) according to manufacturer's instructions of the MegaCult kit. Cytospins of mast cells were stained for tryptase: the cytospin slides were fixed in 1∶3 methanol:acetone and stained overnight at 4°C with 1.8 µg/ml anti-tryptase antibody clone G3 (Chemicon) in TBS (pH = 7.6) with 10% FBS. The next day, the slides were brought to room temperature, washed with TBS, and slides were incubated with polyclonal rabbit anti-mouse immunoglobulins (Dako) for thirty minutes. After another wash, alkaline phosphatase anti-alkaline phosphatase (APAAP) reagent (Dako) was added to the slides for thirty minutes. SigmaFAST (Fast Red TR/Naphthol AS-MX/Levamisol) substrate tablets were used to develop the reaction. Visible color developed on the slides after approximately seven minutes, at which time the reaction was stopped.

## Supporting Information

Figure S1 **Characterization of the developmental potential of one hiPSC line.** FACS analysis of mature cells generated in culture from BliPSC NL-derived progenitors induced with defined factors.
(EPS)
